# Death of the Senior Surgeon to St. George's Hospital

**Published:** 1912-08-31

**Authors:** 


### DEATH OF THE SENIOR SURGEON TO ST. GEORGE'S HOSPITAL.

### A Many-sided Career.

To those who were familiar with his lean, hardy figure and strongly marked weather-beaten features, the announcement of Mr. Clinton Dent's death will come as a most painful surprise. In him the Royal College of Surgeons loses a vice-president, St. George's Hospital a senior surgeon, and the Metropolis a personality in many ways unique. Clinton Thomas Dent was educated at Eton and at Trinity College, Cambridge, whence he entered at St. George's Hospital for his clinical studies. Fortunate in the possession of means which made him independent of his profession, Mr. Dent nevertheless put an energy and a thoroughness into his work and play which seemed to be inexhaustible.

To take first his professional activities as a surgeon, it may be mentioned that Mr. Dent qualified in 1875 and became a Fellow of the Royal College of Surgeons two years later. After holding the post of surgical registrar, he was elected to the staff as assistant surgeon to St. George's Hospital in 1880. In those days promotion was very slow, and Mr. Dent was wont in later years to speak humorously of his fifteen years in the out-patient department, waiting for the vacancy that seemed never to get any nearer. It was in 1895 that he at last obtained beds, and was thus afforded larger opportunities of operating. Although the St. George's staff was singularly healthy in those days, of late years the pendulum has swung the other way. Thus Dr. Lee Dickinson and Mr. Allingham died in harness, Mr. Jones and Mr. Sheild had to resign through impaired health, and Dr. Spriggs has been for several months on the sick list; and now Mr. Dent too is cut off. Besides his work at St. George's, Mr. Dent was for many years on the staff of the Belgrave Hospital for Children, and at the time of his death was consulting surgeon to that hospital. He was for several years a member of the Council of the Royal College of Surgeons, and had lately been re-elected vice-president. He examined for many years at Cambridge University and for the Conjoint Board; and if his aspect was somewhat intimidating to nervous candidates, his consideration and courtesy soon set them at their ease.

In his play Mr. Dent's energy was as remarkable as in his work. Of late years cricket, tennis, and rowing took up most of his leisure; but in the prime of his life mountaineering came an easy first with him. He joined the Alpine Club in 1872, at the age of twenty-one, and was on the committee two years later; secretary in 1878-80, vice-president in 1884, and president 1886-89; the record speaks for itself. The number of his pioneer ascents is large, and it was not only in the Alps but also in the Caucasus that he earned fame amongst his brother climbers. Nor was he content with the ephemeral joys of the actual ascent. From early days an enthusiastic amateur photographer, he photographed his beloved mountains with a success which at that time was phenomenal. His hundreds of beautiful pictures of snow and rock scenery were not merely the successful snapshots of an intrepid climber; they were taken by an artist, whose "composition" was as elegant as his "technique" was masterly.

Mr. Dent wrote a good deal upon mountaineering as well as upon surgical topics; and on everything he did he left the indelible imprint of ripe scholarship and a well-balanced judgment. In 1899 on the outbreak of war he went to Natal as surgeon, and saw much of gunshot wounds which he turned to professional account. So diverse were his interests, that it is difficult to say what were his favourite fields of surgery. Probably amputations were the operations he had most affection for, and his knowledge of all the formal amputations was prodigious and exhaustive. The decay of the formal amputation, consequent on the progress of Listerian surgery, he recognised with regret as inevitable; one of his hobbies was the compilation of the statistics concerning all amputations done by himself and his colleagues at St. George's Hospital. The interest which Mr. Dent took in all forms of trauma and accidental injury was increased when in 1904 he became chief surgeon to the Metropolitan Police. This appointment he filled with a zeal and sympathy which soon made him very popular in the "Force."

Of Mr. Dent's personal qualities much might be written. As an after-dinner speaker he had few superiors in London, both for wit and wisdom in that difficult form of oratory. His house surgeons and dressers at St. George's soon got to know that his epigrammatic criticisms and comments were always kindly meant, however gruff might be the tone and manner of their ejaculation. Indeed, Mr. Dent was one of the kindest hearted of men. His handling of a sick and fractious child was an object-lesson in clinical methods; somehow children seemed to know at once that the bearded, brown-faced, angular, long-necked man was a friend to be trusted. His peculiar gestures and phrases, his petty economies, his wonderful photographs, his cultured scholarship, all these will long be remembered by those who knew him; but most of all his large heart and his devotion to work will survive in the memory of his friends and pupils.

Mr. Dent was in his sixty-second year, and was never married. He died, it is understood, of rapid septicæmia following a septic sore throat; only a fortnight or so before his death he had been playing cricket.

